# The role of mitochondrial genomics in patients with non-alcoholic steatohepatitis (NASH)

**DOI:** 10.1186/s12881-016-0324-0

**Published:** 2016-09-05

**Authors:** Rohini Mehta, Kianoush Jeiran, Aaron B. Koenig, Munkzhul Otgonsuren, Zachary Goodman, Ancha Baranova, Zobair Younossi

**Affiliations:** 1 Betty and Guy Beatty Center for Integrated Research, Inova Fairfax Medical Campus, Falls Church, VA USA; 2 Center for the Study of Chronic Metabolic and Rare Diseases, George Mason University, Fairfax, VA 22033 USA; 3 Betty and Guy Beatty Center for Integrated Research, Claude Moore Center for Research and Education, 3300 Gallows Road, Falls Church, VA 22033 USA

**Keywords:** PNPLA3 rs738409, Hepatic fibrosis, Obesity, Ethnicity

## Abstract

**Background:**

Visceral obesity and metabolic syndrome are commonly associated with non-alcoholic fatty liver disease (NAFLD). The progression of steatosis to NASH depends on a number of metabolic and patient-related factors. The mechanisms of genetic predisposition towards the development of NASH and related fibrosis remain unclear. In this study, our aim was to utilize mitotyping and identify mitochondrial haplotypes that may be associated with NAFLD.

**Methods:**

We examined mitochondrial haplotypes along with patatin-like phospholipase domain containing 3 (PNPLA3) rs738409 genotype to determine their association with NAFLD phenotypes. Whole blood samples were obtained from 341 patients (BMI > 35) undergoing weight reduction surgery after written consent. Liver biopsies were centrally reviewed by a single pathologist based on predetermined pathologic protocol (41.9 % Non-NASH NAFLD, 30.4 % NASH, 27.5 % controls). A 1,122 bp of the mitochondrial control loop was sequenced for each sample and classified into haplogroups.

**Results:**

The presence of haplogroup L exhibits protection against the development of NASH and pericellular fibrosis. The alleles of PNPLA3 locus showed differential distribution in cohorts with NAFLD, NASH and pericellular fibrosis. Heterozygosity at this locus is independently associated with higher risk of having NASH and pericellular fibrosis.

**Conclusion:**

Mitochondrial genetics play an important role in NASH probably by modulation of oxidative stress and the efficiency of oxidative phosphorylation.

**Electronic supplementary material:**

The online version of this article (doi:10.1186/s12881-016-0324-0) contains supplementary material, which is available to authorized users.

## Background

Non-alcoholic fatty liver disease (NAFLD) is a leading cause of chronic liver disease in the United States and worldwide [1, 2]. The prevalence rate of NAFLD is reported between 10 and 35 % in the general population with the prevalence rates being substantially higher in the obese and diabetic individuals [3].

NAFLD is characterized by accumulation of fat, mainly triglycerides, in the absence of excessive alcohol consumption, viral infection, hereditary disorders, or use of steatogenic medications [4, 5]. In its progressive form, NAFLD manifests as non-alcoholic steatohepatitis (NASH) defined as steatosis with ballooning degeneration and/or presence of Mallory-Denk bodies. NASH can ultimately lead to fibrosis followed by cirrhosis and hepatocellular carcinoma (HCC) [6–8].

Both visceral obesity and metabolic syndrome are commonly associated with NASH [9]. However, not all patients with NASH are obese [10]. The progression of steatosis to NASH has been associated with a number of other metabolic factors, including the quality of nutrition and patient’s lifestyle [11] as well as genetic predisposition [12]. The mechanisms of genetic predisposition to the development of NASH remain unclear, but certainly involve the variation in individual susceptibility to NAFLD [12]. A number of nuclear loci have already been identified as potential contributors to development of NASH [13].

In 2008, a genome-wide association study in a population comprising Hispanic, African American and European American subjects showed that a common genetic variant in the patatin-like phospholipase domain containing 3 protein (PNPLA3) gene is associated with liver fat accumulation [14]. The isoleucine to methionine substitution at position 148 in the patatin-like phospholipase domain containing 3 protein (PNPLA3; I148M variant, rs738409) has since then been shown to be associated with liver fat accumulation and severity of NAFLD [15–18]. The interaction between PNPLA3 and BMI has been supported by several population based studies [19–21]. The studies have shown that body mass index interacts with PNPLA3 and increases susceptibility to NAFLD. However, PNPLA3 function and the effect of the I148M substitution are not yet completely understood.

PNPLA3 protein plays anabolic and catabolic role in lipid metabolism. Purified PNPLA3 protein has been shown to have both hydrolase and lysophosphatidic acid transacetylase activities, with the hydrolase activity predominating [22–24]. However, its in-vivo function and physiological relevance remain controversial. In-vitro studies have shown the 148 M mutation to be a loss-of-function mutation abolishing the hydrolase activity [22, 24] while 148 M mutation is a gain of function mutation for lysophosphatidic acid transacetylase (LPAAT) activity [23]. Consistent with a hydrolase activity hypothesis, a relative reduction in hepatic very low-density lipoprotein secretion with the 148 M mutation has been shown in vitro and in humans [25]. Contrary to the hydrolase activity, wild-type PNPLA3 was also found to have lysophosphatidic acid transacetylase activity and the 148 M mutation was found to be a gain of function of the lipogenic activity [26]. In agreement, rats on a high-fat diet treated with pnpla3 antisense oligonucleotides showed decreased fatty acid esterification into hepatic triglycerides and reduced insulin resistance [27]. In a recent study of obese adolescents with hepatic steatosis, the I148M variant has been shown to modulate the association between oxidized metabolites derived from linoleic acid and cytokeratin 18 fragment, a robust biomarker of liver injury [28]. Recently, a cell specific PNPLA3 function linking PNPLA3 to liver fibrosis has been shown [29, 30]. The authors show retinyl-palmitate lipase activity of PNPLA3 in human hepatic stellate cells with I148M being a loss of function mutation. Thus, the genotypic effect of PNPLA3 protein is complex.

In this study, our central hypothesis is that mitochondrial haplotypes along with a susceptible nuclear genetic background may be involved in predisposing to NAFLD and NASH. With this in mind, we examined mitochondrial haplotypes along with patatin-like phospholipase domain containing 3 (PNPLA3) rs738409 genotype and determined their association with NAFLD and NASH.

## Methods

### Patient cohort

After informed written consent, blood samples were obtained from 341 patients (BMI > 35) undergoing weight reduction surgery and immediately flash frozen in liquid nitrogen followed by storage in −80 °C. All patients had routine liver biopsies taken which were read by a central pathologist. Clinical data from the time of enrollment were collected and de-identified in compliance with HIPAA regulations. This study has been approved by Internal Review Board of Inova Fairfax Hospital (Federal Assurance FWA00000573).

For the purpose of this study, metabolic syndrome was defined by the presence of three or more of the five following features: total cholesterol ≥200 mg/dl of blood, LDL ≥130 mg/dl, triglyceride ≥150 mg/dl, HDL < 50 mg/dl in women or less than 40 mg/dl in men, and BMI >30. None of the included subjects reported to have excessively consumed alcohol (>10 g/day in women and >20 g/day in men) in the past 5 years. Other chronic liver diseases were excluded by negative serology for hepatitis B and C, no history of toxic exposure and no other cause of chronic liver disease. Ethnicity was recorded as self-reported.

### Liver biopsy

Liver biopsies were centrally reviewed by a single pathologist based on predetermined grading system [31]. Histological features such as portal inflammation, lymphoplasmacytic lobular inflammation, polymorphonuclear lobular inflammation, Kupffer cell hypertrophy, apoptotic bodies, focal parenchymal necrosis, glycogen nuclei, hepatocellular ballooning, and Mallory-Denk bodies were evaluated in the H & E sections. Steatosis (Non-NASH NAFLD) was defined as fat with/without lobular inflammation and/or portal inflammation. The severity of steatosis was evaluated according to the percentage of liver parenchyma occupied by fat: 0 = none, 1 ≤ 5 %, 2 = 6–33 %, 3 = 34–66 %, and 4 > 66 %. Non-alcoholic steatohepatitis (NASH) was defined as steatosis, lobular inflammation, and ballooning degeneration with or without Mallory Denk bodies, and with or without fibrosis. Severity of pericellular and portal fibrosis was determined by Masson trichome staining of the biopsy. The scoring was as follows: 0 = no fibrosis, 1 = mild fibrosis, 2 = moderate fibrosis, 3 = marked fibrosis. A score of ≥ 3 was considered as advanced hepatic fibrosis and a score of <3 as mild/no hepatic fibrosis. Patients with hepatic steatosis or NASH were considered to have NAFLD. Patients with normal liver histology were considered as controls.

### Isolation of total DNA

Total DNA was extracted from whole blood cells using QIAamp® kits in accordance with manufacturer’s instructions (Qiagen, USA). DNA was then quantified and quality assessed by spectrophotometer (GeneQuant 1300, General Electric). Additionally, to assess the integrity of extracted DNA, agarose gel electrophoresis was carried out. The gel was inspected for evidence of poor DNA quality visible as degradation/smearing. Each DNA sample was diluted 1:10 prior to its use as PCR template.

### Primer design and PCR

Amplification of the 1,122 base pair region of the mitochondrial control loop was achieved by using 4 pairs of primers (Additional file 1: Table S1) with 100 basepair overlap of resultant amplicons.

The PCR reaction was set up for 25 μl, containing 12.5 μl HotStar Taq Master Mix Kit (Qiagen®), 2.5 μl (250 nM) of each primer, 1 μl of 1:10 diluted DNA, and 6.5 μl molecular grade water. The thermal cycling was as follows: initial denaturation at 95 °C for 15 min; 30 cycles of 95 °C for 1 min; annealing for 1 min; 72 °C for 1 min. For sequencing, 20 μl of each PCR product was sent for Sanger sequencing to GenScript (Piscataway, NJ).

### Sequence assembly and mitochondrial haplotypes identification

Chromatograms of forward and reverse fragments for each primer pair were examined using Chromas Lite® (Technelysium, Brisbane, Australia). The assembly and editing of the fragments was carried out using Sequencher® (version 5.2.4), a consensus sequence of 1,122 bp of the mitochondrial control loop was generated for each DNA sample. All DNA sequences were uploaded into MitoTool software [32] and classified into 15 haplogroups. The success rate of sequencing and subsequent alignment was 96 %.

### PNPLA3 SNP rs738409 genotyping

Genotyping for the PNPLA3 SNP rs738409 was completed using predesigned TaqMan probe (Applied Biosystems, CA). Genotyping of rs738409 (C_7241_10) was carried out on CFX96 PCR instrument (Biorad, USA) according to the manufacturer’s protocol (50 °C for 2 min, 95 °C for 10 min, and then 40 cycles of 95 °C for 15 s and 60 °C for 1.5 min). After PCR amplification, a post-PCR plate reads were carried out to generate allelic discrimination plot. The allele assignments were independently verified by RFLP analysis [33].

### Statistical analysis

To assess the association of SNP rs738409 genotype and mitochondrial haplotypes, a number of statistical procedures were employed. The pairwise comparison cohorts consisted of subjects with normal liver histology (controls), Non-NASH NAFLD, NASH and NAFLD with pericellular fibrosis. For continuous variables, *p* values were determined by *t*-test and for categorical variables, *p* values were determined by chi-square test. Linear regression models were tested to assess the association between genotype and continuous phenotypes, and logistic regression models were used to test association between genotype and categorical phenotype. Multivariate analysis was carried out to determine independent predictors of NAFLD.

## Results

The clinical, demographic and biochemical characteristics of study subjects (*N* = 341; BMI =48 ± 9.13, Age = 44 ± 11.2, 41.9 % Non-NASH NAFLD, 30.4 % NASH, 27.5 % controls) are summarized in Table 1.

**Table 1:** Clinical and demographic characteristic of Study Cohort

Demographic/Clinical Variable	Average ± SD or N (%)
Age (yrs)	44 ± 11.2
Males	88 (25 %)
BMI (kg/m^2^)	48 ± 9.13
ALT (U/L)	34.59 ± 25.9
AST (U/L)	26.37 ± 19.35
Glucose (mg/dl)	108.8 ± 36.76
Triglycerides (mg/dl)	157.9 ± 93.45
Total cholesterol (mg/dl)	187.69 ± 39.3
HDL (mg/dl)	47.28 ± 12.85
LDL (mg/dl)	108.2 ± 35.8
Normal liver biopsy	94 (27.5 %)
Non-NASH NAFLD	143 (41.9 %)
Histologic NASH	104 (30.4 %)

Continuous variables are represented as mean ± standard deviation (SD), and categorical variables as percentages

Patients with histologic NASH when compared to non-NASH NAFLD had higher levels of serum aminotransferases compared to patients with non-NASH NAFLD (*p* < 0.001) (Table 2). In contrast, fasting serum HDL and platelets were lower in NASH patients compared to those with non-NASH NAFLD (*p* < 0.001) (Table 2). Fasting serum glucose, serum aminotransferases, serum triglycerides and total cholesterol was higher, while serum HDL and platelets were lower (*p* < 0.001) (Table 2) in patients with Non-NASH NAFLD compared to controls with normal liver histology. Among the NASH and non-NASH NAFLD subjects, there was no significant difference in the mean BMI, age, serum triglycerides, serum cholesterol, serum LDL and fasting glucose levels (*p* > 0.05) (Table 2). Similarly, subjects with normal liver histology (controls) had no significant difference in the mean BMI, WBC, platelets, serum LDL and HDL levels when compared to Non-NASH NAFLD subjects (*p* > 0.05) (Table 2). Notably, patients with normal liver histology were more likely to be younger and had higher platelets counts when compared to those with NASH (*p* < 0.001) (Table 2).

**Table 2:** Comparisons of Controls (normal liver histology) with Non-NASH NAFLD and NASH

Variable	Normal liver histology (*N* = 94)	Non-NASH NAFLD (*N* = 143)	NASH (*N* = 104)	*P*-value*	*P*-value†	*P*-value‡
Age (yrs)	41.48 ± 10.86	44.08 ± 11.39	46.42 ± 10.94	0.0814	**0.0017**	0.1053
BMI (kg/m^2^)	46.80 ± 7.98	47.80 ± 8.50	49.47 ± 10.56	0.3733	0.0512	0.1715
WBC (x 10^3^/uL)	7.81 ± 2.54	8.08 ± 2.21	8.82 ± 8.81	0.3784	0.2855	0.3366
Platelets (x 10^3^/uL)	303.51 ± 69.73	291.01 ± 64.23	268.69 ± 66.97	0.1632	**0.0005**	**0.0094**
Insulin Resistance	1.92 ± 2.83	2.40 ± 1.18	8.37 ± 11.00	0.4879	**0.0413**	**0.0154**
Glucose (mg/dL)	94.16 ± 21.74	110.47 ± 36.20	120.22 ± 43.50	**0.0002**	**<.0001**	0.0641
ALT (U/L)	22.20 ± 9.74	31.58 ± 16.35	49.51 ± 37.25	**<.0001**	**<.0001**	**<.0001**
AST (U/L)	19.41 ± 5.57	23.49 ± 11.04	36.43 ± 29.69	**0.0013**	**<.0001**	**<.0001**
Triglycerides (mg/dL)	125.84 ± 58.42	160.26 ± 87.31	182.10 ± 117.04	**0.0018**	**0.0001**	0.1152
LDL (mg/dL)	100.96 ± 35.75	110.75 ± 35.40	111.71 ± 36.34	0.0620	0.0646	0.8555
Total cholesterol (mg/dL)	175.84 ± 34.00	193.24 ± 39.40	190.10 ± 41.96	**0.0011**	**0.0150**	0.5683
HDL (mg/dL)	50.40 ± 12.42	48.58 ± 13.63	42.68 ± 10.96	0.3461	**<.0001**	**0.0014**
White: N (%)	66 (70.2 %)	112 (78.3 %)	84 (80.8 %)	0.1579	0.0835	0.6389
Diabetes: N (%)	17 (18.3 %)	44 (30.8 %)	46 (44.2 %)	**0.0322**	**<.0001**	**0.030**
Hyperlipidemia: N (%)	34 (37.4 %)	69 (50.0 %)	59 (59.6 %)	0.0599	**0.0022**	0.1438
Hypertension: N (%)	40 (44.4 %)	81 (57.9 %)	64 (65.3 %)	**0.0468**	**0.0041**	0.2464
Sleep Apnea: N (%)	53 (57.6 %)	100 (71.9 %)	81 (80.2 %)	**0.0241**	**0.0007**	0.1425

*Pairwise comparison between controls (normal liver histology) and Non-NASH NAFLD cohorts; †Pairwise comparison between controls (normal liver histology) and NASH cohorts; ‡Pairwise comparison between Non-NASH NAFLD and NASH cohorts. Continuous variables are represented as mean ± standard deviation (SD), and categorical variables as percentages. For convenience, all statistically significant *p*-values are highlighted by bold font

### Both NAFLD and NASH are associated with PNPLA3 rs738409 variants

In previous studies, PNPLA3 variant I148M (rs738409) has been associated with both the obese phenotype and NAFLD. In order to assess any potential interaction between PNPLA3 and mitochondrial DNA, we genotyped rs738409 in all of our patients. The prevalence of the homozygous CC allele in study population was 58 %, while GG allele frequency was 7.5 % and GC allele frequency was 33.8 %. For the PNPLA3 SNP rs738409 alleles, observed frequencies were in accordance with Hardy-Weinberg equilibrium [34–36].

**Fig. 1:**
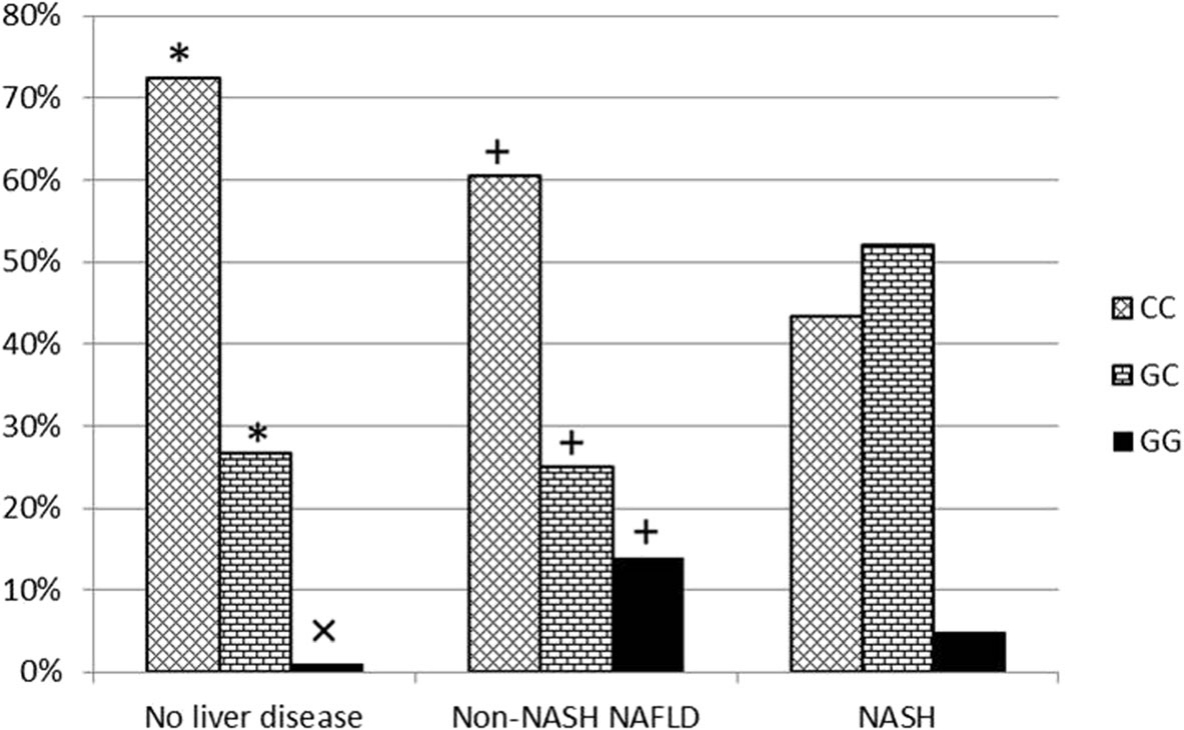
The prevalence of PNPLA3 rs738409 variant in NASH, Non-NASH NAFLD and Normal Liver Histology cohorts (*p* < 0.05 significant). * Significant pair-wise comparison between controls with normal liver histology and NASH; + Significant pair-wise comparison between Non-NASH NAFLD and NASH; **x** Significant pair-wise comparison between controls with normal liver histology and Non-NASH NAFLD

In controls, the CC genotype of PNPLA3 rs738409 was more prevalent than in the NASH cohort (72.3 vs 43.3 %, *p* < 0.001). Similarly, the CC genotype was more common in patients with non-NASH NAFLD as compared to NASH (60.8 vs 43.3 %, *p* = 0.006), while the prevalence GC genotype was higher in patients with NASH as compared to those with Non-NASH NAFLD (51.9 vs 25.5 %, *p* < 0.0001) and higher as compared with the controls (51.9 vs 26.6 %, *p* = 0.0003). Interestingly, the homozygosity for the risk allele (GG genotype) had higher prevalence in Non-NASH NAFLD patients as compared to NASH patients (14 vs 4.8 %, *p* = 0.018) (Fig. 1).

### L mitochondrial haplogroup and NAFLD

In our study cohort, six common mitochondrial haplogroups (H, L, U, K, J and T) had a prevalence of greater than 5 %. For the purpose of further analysis, all the uncommon (<5 %) haplotypes (A, C, I, W, M, N, X, B and R) were pooled together into one group (“Other”). Among the self-reported ethnic groups, 77 % were White, 14.7 % were African Americans, 3.5 % were Hispanic (Additional file 1: Figure S1).

**Fig. 2:**
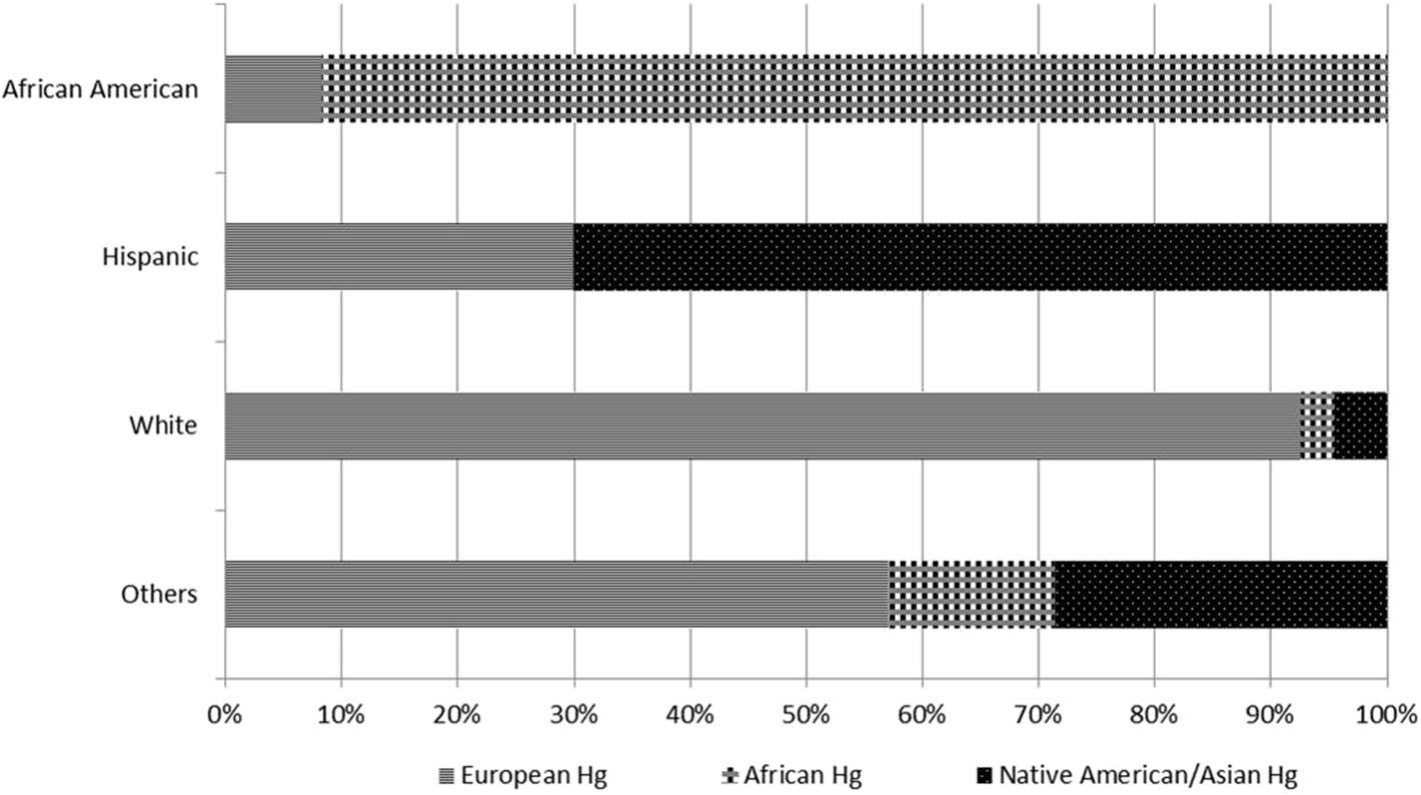
Haplogroup distribution by self-identified ethnicity in our study population. European lineages: H, T, U, V, X, K, N, I, J, HV, W; African Lineages: L, L1, L2, L3, L3; Asian/American Indian: A, B, F, macro-haplogroup M (C, D, E, and G), X

The overall distribution of haplogroup lineages within the self-identified race/ethnicity cohorts was in agreement with a recent survey of haplogroups in United States [37]. For example, in self-identified population of whites, European lineage haplogroups were the most common haplogroup (35.5 %), with haplogroup H (41 %) being the most common, followed by haplogroups U (17 %), K (12 %), J (11 %) and T (9 %). These data are in agreement with the H frequency reported recently to be 43 % in non-hispanic whites of United States [37]. Among self-identified African Americans, 91 % were confirmed as being of African lineages (Fig. 2) with L2 (40 %) being the most common haplogroup followed by L3 (36 %). Among self-identified Hispanics, 70 % were confirmed as of Asian/American Indian lineage (Fig. 2).

Mitochondrial haplogroup assessment showed that African lineage Haplogroup L (encompasses L0, L1, L2 and L3 subtypes) was less prevalent in Non-NASH NAFLD and NASH cohorts as compared to the cohort with normal liver histology, *p* < 0.018 and *p* < 0.0002, respectively. This suggests that L haplogroup may protect against NAFLD (Table 3).

**Table 3:** Distribution of mitochondrial haplotypes in study groups

Variable	Normal liver histology (*N* = 94)	Non-NASH NAFLD (*N* = 143)	NASH (*N* = 104)	*P* value*	*P*-value†	*P* value‡
Haplotype						
H	28 (29.8 %)	56 (39.2 %)	37 (35.6 %)	0.1400	0.3863	0.5660
L	23 (24.5 %)	22 (15.4 %)	6 (5.8 %)	0.0811	**0.0002**	**0.0186**
U	11 (11.7 %)	14 (9.8 %)	17 (16.3 %)	0.6393	0.3490	0.1247
K	8 (8.5 %)	12 (8.4 %)	11 (10.6 %)	0.9743	0.6221	0.5595
J	11 (11.7 %)	11 (7.7 %)	5 (4.8 %)	0.2980	0.0755	0.3631
T	6 (6.4 %)	9 (6.3 %)	6 (5.8 %)	0.9780	0.8566	0.8647
Other	4 (4.3 %)	13 (9.1 %)	18 (17.3 %)	0.1582	**0.0035**	0.0543

*Pairwise comparison between controls (normal liver histology) and Non-NASH NAFLD cohorts; †Pairwise comparison between controls (normal liver histology) and NASH cohorts; ‡Pairwise comparison between Non-NASH NAFLD and NASH cohorts. For convenience, significant *p*-values are highlighted by bold font

### Nuclear and mitochondrial genotypes as NASH predictors

An initial univariate analysis showed that in population of patients with NAFLD (*N* = 247), the heterozygosity for PNPLA3 rs738409 variant may serve as independent predictor of having histologic NASH (OR 2.9: 95 % CI 1.67 – 5.05). When similar analyses were performed for mitochondrial haplogroups, only L haplogroup provided protection against histologic NASH (OR 0.34: 95 % CI 0.13 – 0.86) (Table 4).

**Table 4:** Univariate-adjusted analysis for NASH vs Non-NASH NAFLD

Variable Name	Sample Size	*P* Value	Odds Ratio	Lower OR	Upper OR
Age (yrs)	247	0.106	1.02	1.00	1.04
BMI (kg/m^2^)	244	0.173	1.02	0.99	1.05
WBC (x 10^3^/uL)	246	0.396	1.02	0.97	1.08
Platelet (x 10^3^/ul)	242	**0.011**	0.99	0.99	1.00
IR (HOMA)	40	0.009	2.20	1.22	3.97
Glucose (mg/dL)	232	0.068	1.01	1.00	1.01
ALT (U/L)	245	**0.000**	1.03	1.02	1.05
AST (U/L)	245	**0.000**	1.05	1.03	1.08
Triglycerides (mg/dL)	219	0.127	1.00	1.00	1.01
LDL (mg/dL)	193	0.854	1.00	0.99	1.01
Total Cholesterol (mg/dL)	223	0.567	1.00	0.99	1.00
HDL (mg/dL)	198	**0.002**	0.96	0.94	0.99
Male	247	**0.000**	4.47	2.48	8.06
White	247	0.639	1.16	0.62	2.18
Diabetes	247	**0.031**	1.78	1.06	3.02
Hyperlipidemia	237	0.144	1.47	0.88	2.49
Hypertension	238	0.247	1.37	0.80	2.34
Haplotype,					
H	247	0.566	0.86	0.51	1.45
L	247	**0.023**	0.34	0.13	0.86
U	247	0.128	1.80	0.84	3.84
Other	247	0.058	2.09	0.98	4.49
K	247	0.560	1.29	0.55	3.05
J	247	0.367	0.61	0.20	1.80
T	247	0.866	0.91	0.31	2.65
PNPLA3 SNP,					
CC	Reference				
GC	247	**0.000**	2.90	1.67	5.05
GG	247	0.172	0.48	0.17	1.37

For convenience, significant *p*-values are highlighted by bold font

In non-L haplotype cohort, further multivariate analysis after adjusting for gender, age and ALT showed that patients with GC genotype of PNPLA3 locus are twice more likely to have NASH as compared to those with genotype CC (OR 2.66: 95 % CI 1.43 – 4.95).

### Nuclear and mitochondrial genotypes in pericellular fibrosis

Since fibrosis is an important predictor of clinical outcome, we assessed the association between mitochondrial haplogroups and PNPLA3 genotype with pericellular fibrosis as well as with other histologically features.

NAFLD patients without pericellular fibrosis (*N* = 247) had higher platelet and serum HDL levels (Table 5) compared to those with pericellular fibrosis (*N* = 95). Serum aminotransferases were lower in these subjects compared to those with pericellular fibrosis. Notably, NAFLD patients with pericellular fibrosis were enriched for L haplogroup (L0, L1, L2, L3) (15.8 vs 4.2 %, *p* = 0.005), compared to NAFLD patients without pericellular fibrosis.

**Table 5:** Comparisons of NAFLD patients with pericellular fibrosis with NAFLD patients without pericellular fibrosis

Variable	NAFLD without	NAFLD with	*P*-value
	p. fibrosis (*N* = 152)	p. fibrosis (*N* = 95)	
Age (yrs)	44.03 ± 11.08	46.73 ± 11.35	0.0609
BMI (kg/m^2^)	48.05 ± 8.94	49.23 ± 10.19	0.1752
WBC (x 10^3^/uL)	8.16 ± 2.35	8.77 ± 9.15	0.5668
Platelets (x 10^3^/uL)	290.32 ± 63.79	267.63 ± 67.91	**0.0094**
Insulin Resistance	2.42 ± 1.16	8.69 ± 11.25	**0.0009**
Glucose (mg/dL)	111.01 ± 36.85	120.25 ± 43.34	0.2630
ALT (U/L)	32.38 ± 17.62	49.95 ± 37.96	**<.0001**
AST (U/L)	23.97 ± 11.78	36.88 ± 30.51	**<.0001**
Triglycerides (mg/dL)	159.48 ± 86.89	185.17 ± 119.26	0.0760
LDL (mg/dL)	112.12 ± 36.03	109.52 ± 35.33	0.7968
Total cholesterol (mg/dL)	193.96 ± 39.81	188.62 ± 41.43	0.3486
HDL (mg/dL)	48.38 ± 13.47	42.69 ± 11.20	**0.0004**
Male: N (%)	26 (17.1 %)	45 (47.4 %)	**<.0001**
White: N (%)	119 (78.3 %)	77 (81.1 %)	0.6017
HAPLOTYPE, N (%)			
H	58 (38.2 %)	35 (36.8 %)	0.8355
L	24 (15.8 %)	4 (4.2 %)	**0.0052**
U	17 (11.2 %)	14 (14.7 %)	0.4123
Other	13 (8.6 %)	18 (18.9 %)	**0.0164**
K	12 (7.9 %)	11 (11.6 %)	0.3324
J	11 (7.2 %)	5 (5.3 %)	0.5398
T	10 (6.6 %)	5 (5.3 %)	0.6736
PNPLA3_SNP, N (%)			
CC	94 (61.8 %)	38 (40.0 %)	**0.0008**
GC	38 (25.0 %)	52 (54.7 %)	**<0.0001**
GG	20 (13.2 %)	5 (5.3 %)	**0.0454**

For convenience, significant *p*-values are highlighted by bold font

Homozygous genotypes for PNPLA3: CC and GG, were more prevalent in NAFLD patients without pericellular fibrosis (61.8 vs 40 % for CC, *p* = 0.0008 and 13.2 vs 5.3 %, *p* = 0.04). However, the association of GG genotype with pericellular fibrosis should be considered with caution due to the small sample size of GG genotype. GC genotype, on the other hand was more common in pericellular fibrosis cohort (54.7 vs 25 %, *p* < 0.001).

Univariate analysis revealed that L haplotype was protective against odds of developing pericellular fibrosis (OR 0.23: 95 % CI 0.08–0.70) and patients with heterozygosity in PNPLA3 locus had a 3 times higher odds of developing pericellular fibrosis as compared to patients CC genotype (OR 3.39: 95 % CI 1.93 – 5.94) (Table 6). After adjusting for age, number of platelets, levels of ALT, AST, triglycerides, HDL and male gender, individuals with PNPLA3 GC genotype remained associated with higher likelihood of having pericellular fibrosis than those with PNPLA3 genotype CC (OR 4.38: 95 % CI 2.17 – 8.85).

**Table 6:** Univariate-adjusted analysis for Pericellular fibrosis

Variable Name	Sample Size	*P* Value	Odds Ratio	Lower OR	Upper OR
Age (yrs)	247	0.067	1.02	1.00	1.05
BMI (kg/m^2^)	244	0.345	1.01	0.99	1.04
WBC (x 10^3^/uL)	246	0.467	1.02	0.97	1.07
Platelet (x 10^3^/ul)	242	**0.011**	0.99	0.99	1.00
IR (HOMA)	40	0.008	2.26	1.24	4.14
Glucose (mg/dL)	232	0.087	1.01	1.00	1.01
ALT (U/L)	245	**0.000**	1.03	1.01	1.04
AST (U/L)	245	**0.000**	1.05	1.02	1.07
Triglycerides (mg/dL)	219	0.080	1.00	1.00	1.01
LDL (mg/dL)	193	0.623	1.00	0.99	1.01
Total Cholesterol (mg/dL)	223	0.338	1.00	0.99	1.00
HDL (mg/dL)	198	**0.003**	0.96	0.94	0.99
Male	247	**0.000**	4.36	2.43	7.82
White	247	0.602	1.19	0.62	2.25
Diabetes	247	**0.011**	1.99	1.17	3.38
Hyperlipidemia	237	**0.012**	1.99	1.16	3.42
Hypertension	238	0.254	1.37	0.80	2.36
Haplotype					
H	247	0.836	0.95	0.56	1.61
L	247	**0.009**	0.23	0.08	0.70
U	247	0.414	1.37	0.64	2.93
Other	247	**0.019**	2.50	1.16	5.38
K	247	0.335	1.53	0.65	3.62
J	247	0.541	0.71	0.24	2.12
T	247	0.674	0.79	0.26	2.38
PNPLA3_SNP					
CC	Reference				
GC	247	**0.000**	3.39	1.93	5.94
GG	247	0.370	0.62	0.22	1.77

For convenience, significant *p*-values are highlighted by bold font

## Discussion

Oxidative stress is an important player in development of progressive NAFLD or NASH. In fact, oxidative stress related cellular damage, reflected as hepatic necrosis or apoptosis, is a mitochondrial dependent factor that potentially influences the progression of NAFLD [38–40]. Given that mitochondria are critical players in maintaining balance of energy input and expenditure [41] as well as the pathogenesis of NASH, it is enticing to explore mitochondrial genome variation as a potential genetic factor that could contribute to the pathogenesis of NASH.

Mitochondria have their own circular DNA that can be categorized into various haplogroups based on variants within its non-coding hypervariable region. Notably, the non-coding region is indispensable for replication and transcription of the mitochondrial genome. Thus variations in this region are expected to influence the ability of mitochondria to produce ATP and ROS. In turn, availability of ATP and the production of ROS are known to affect tissue responses to extrinsic or intrinsic stressors and energy demands. Consequently, mitochondrial DNA variations have been the focus of studies in patients with altered metabolism [42–44]. Additionally, in a recent study, there is evidence that mitochondrial DNA haplogroups may influence expression of nuclear genes that are induced under oxidative stress [45].

NAFLD is a complex disease with genetic and environmental factors interacting to influence the development and progression of the disease. The relative importance of these factors may vary between populations depending on genetic background and lifestyle. This study is unique as we show for the first time the differential prevalence of mitochondrial haplogroups in obese patients (BMI > 35) with varying severity of NAFLD. We also examine the interaction between mitochondrial genome variation and nuclear PNPLA3 genotype in this patient cohort.

In this study, we present evidence that mitochondrial haplogroup L is less prevalent in subjects with NASH and may thus be protective against NASH (Table 3). Haplogroup L has the highest sequence divergence from other haplogroups [46]. A recent study demonstrated that L haplogroup is also functionally different [45]. When compared to the cybrids with H haplogroup, those with L haplogroup demonstrated lower levels of the ROS production but similar levels of ATP generation, indicative of more efficient oxidative phosphorylation with lower electron leakage and higher efficiency of aerobic respiration [45]. These observations support the hypothesis that the patients with L haplogroup may be intrinsically protected from oxidative stress and thus, potential liver damage by ROS.

It is also important to note that the mitochondrial haplogroups are commonly used as a proxy to ethnic origin of individual. According to the population-based studies, NAFLD and NASH are more prevalent in individuals of Hispanic origin [3, 47], while African Americans are somewhat protected against high prevalence of NAFLD and NASH [48]. The high prevalence of L haplogroup in subjects of African origin may serve as a physiological explanation for this phenomenon, although further studies will be needed to determine the causality. In our study cohort, 88 % (*N* = 44) of the self-reported African Americans had L haplotype and 66 % (*N* = 33) of them had normal liver biopsy, 22 % (*N* = 11) had steatosis and 12 % (*N* = 6) had NASH. Additional studies with a larger African American cohort will be needed to further validate this trend. Notably, the haplotype results of our study are consistent with the haplogroup proportions reported in a recent large population based study in United States [37].

In addition to mitochondrial haplotypes, we also measured the well-described gene PNPLA3 variant rs738409 associated with NAFLD. The rs738409 variant is located in coding region of PNPLA3 gene. This variant corresponds to Isoleucine to Methionine substitution (p.I148M) and has previously been identified as risk factor for steatohepatitis, NAFLD-cirrhosis [14, 49] and hepatocellular carcinoma [15, 20, 50–52]. The 148 M variant has also been linked to elevated aminotransferase levels [53] and liver fibrosis [54]. However, the effect of the I148M substitution on the functioning of PNPLA3 is still enigmatic [15]. The 148 M variation is reported to result in loss of hydrolase activity (loss-of-function model) [22, 24, 55] and thus cause accumulation of triglyceride in the liver. However, studies in knock-out mice show contrary results [26, 56]. These studies show that loss of PNPLA3 hydrolase activity does not alter hepatic triglycerides. PNPLA3 variant has also been shown to have a gain of lysophosphatidic acid transacetylase (LPAAT) activity. The 148 M substitution increases LPAAT activity and results in increased TAG synthesis (gain-of-function model) [15, 23]. Thus, PNPLA3 148 M variant may increase hepatic triglyceride levels by multiple changes in triglyceride metabolism.

Our results with regards to PNPLA3 are in agreement with the previously reported studies. The alleles of PNPLA3 locus showed differential distribution in cohorts with NAFLD, NASH (Table 2) and pericellular fibrosis. Moreover, multivariate analysis showed that heterozygosity in this locus is independently associated with higher risk of having NASH as well as with pericellular fibrosis. Overall, carrying G allele in position rs738409 appears to increase the odds of developing NASH and pericellular fibrosis. Importantly, in the final multivariate model of NAFLD (Table 4), no interaction between the nuclear rs738409 variant and mitochondrial haplogroup was detected. This indicates that the somatic genotype and mitochondrial genotype can have independent and important impact in the pathogenesis of NASH.

## Conclusion

In conclusion, our data shows that the presence of haplogroup L exhibits certain degree of protection against the development of NASH and pericellular fibrosis. This suggests that, besides nuclear genome variants and environmental factors, the mitochondrial genetics may play an important role in NAFLD probably by modulation of oxidative stress and the efficiency of oxidative phosphorylation. Further studies will be needed to confirm the causality. Moreover, United States cohorts are substantially admixed, resulting in ambiguity in the questionnaire-based ethnicity assessments [57]. Thus, ethnicity verifying mitochondrial haplogroup data should be coupled with the other biomarkers to serve as a valuable resource for future studies investigating NAFLD and NASH in such populations.

## Additional file

Additional file 1:
**Table S1** Forward and reverse primer sequences for mitochondrial control region amplification. **Figure S1** Self-reported ethnicity distribution in the study cohort. (DOCX 47 kb)

## Abbreviations

ALT: Alanine transaminase
AST: Aspartate aminotransferase
ATP: Adenosine triphosphate
BMI: Body mass index
DNA: Deoxyribonucleic acids
HDL: High density lipoproteins
LDL: Low density lipoproteins
NAFLD: Non-alcoholic fatty liver disease
NASH: Non-alcoholic steatohepatitis
PCR: Polymerase chain reaction
PNPLA3: patatin-like phospholipase domain containing 3
ROS: Reactive oxygen species
SNP: Single nucleotide polymorphism
