# Supplementary material for: The role of mitochondrial genomics in patients with non-alcoholic steatohepatitis (NASH)
Source: BMC Med Genet. 2016 Sep 5;17(1):63. doi: 10.1186/s12881-016-0324-0 (PMC5011877; doi:10.1186/s12881-016-0324-0)

**Supplementary Table 1:** Forward and reverse primer sequences for mitochondrial control region amplification.

| Region | Sequence | Amplicon Length | Annealing temperature | Reference |
| --- | --- | --- | --- | --- |
| H16401  L15997 | 5′-TGATTTCACGGAGGATGGTG-3′  5′-CACCATTAGCACCCAAAGCT-3′ | 443bp | 50 ^0^C | Orekhov et al., 1999 |
| H119  L16201 | 5'-ACATAGGGTGCTCCGGCT-3'  5'-ACAAGCAAGTACAGCAATCAACC-3' | 489bp | 55 ^0^C | Designed |
| H340  L073 | 5'-CTGTTAAAAGTGCATACCGCCA-3'  5'-CTCACGGGAGCTCTCTCCATGC-3' | 401bp | 50 ^0^C | Orekhov et al., 1999 |
| H574  L438 | 5'-GGTGATGTGAGCCCGTCTAA-3'  5'-GCTTCTGGCCACAGCACTTA-3' | 323bp | 50 ^0^C | Wang et al., 2013 |

**Supplementary Figure 1:** Self-reported ethnicity distribution in the study cohort


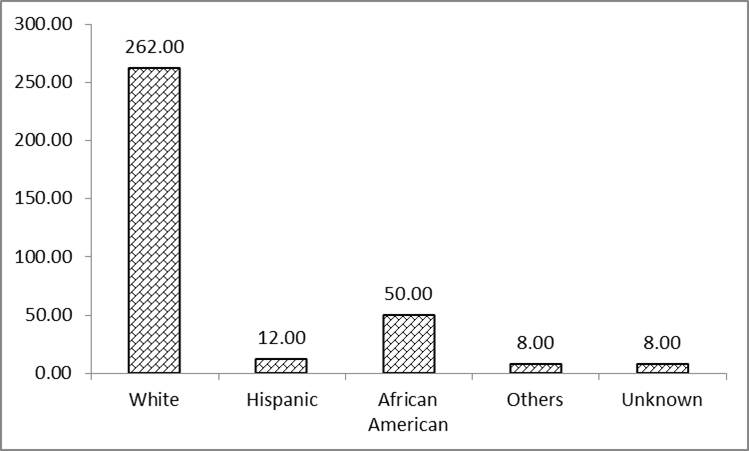

Supplement: Additional file 1: — Table S1 Forward and reverse primer sequences for mitochondrial control region amplification. Figure S1 Self-reported ethnicity distribution in the study cohort. (DOCX 47 kb) [file 12881_2016_324_MOESM1_ESM.docx]
